# Scalable Room‐Temperature Terahertz Graphene Cameras

**DOI:** 10.1002/smtd.70657

**Published:** 2026-04-27

**Authors:** Lili Shi, Leonardo Viti, Miriam S. Vitiello

**Affiliations:** ^1^ CNR‐Istituto Nanoscienze and NEST‐ Scuola Normale Superiore Pisa Italy

## Abstract

Terahertz (THz) imaging has emerged as a powerful tool for non‐destructive, label‐free analysis across several scientific disciplines, ranging from materials science to biomedical research. By capturing the spatial‐dependent information in a broad range of frequencies, this technique enables the identification of chemical composition, structural heterogeneity, and dynamic processes in complex samples. However, the practical deployment of hyperspectral or broadband THz imaging has been limited by the performance, cost, and scalability of conventional detector technologies. In this work, we present two architectures of cameras based on large‐area arrays of graphene field‐effect transistors (GFETs), operating at room temperature, in a broadband configuration, and at terahertz frequencies. The devised GFET‐based detectors exploit the photo‐thermoelectric effect and are optimized for wideband sensitivity through on‐chip broadband antenna design and gate‐tunable responsivity. We thoroughly characterize the performance of individual pixel elements and introduce optimized multiplexed readout circuitry tailored for each configuration. Our GFET‐based THz cameras exhibit exceptional performance metrics, including a noise equivalent power (NEP) on the order of nanowatts per square root hertz (nW/Hz^1/2^) and maximum responsivities reaching 36 V/W. Additionally, the pixel‐to‐pixel uniformity in sensitivity and electrical characteristics significantly reduces the complexity of calibration and control, enabling scalable implementations for large‐format arrays.

## 1 Introduction

The terahertz (THz) frequency range of the electromagnetic spectrum (1–10 THz frequency, 300–30 µm wavelength) has stimulated large interest over the past years for the development of compact imaging technologies to be adopted in a broad range of application fields, including process and quality control [1, 2], intricate nut inspection [3], security screening [4], gas and molecular spectroscopy [5, 6], medical and biological imaging [7, 8], sensing [9], quantum technologies [10], and precise cultural heritage diagnosis [11]. Unlike conventional imaging modalities, each plagued by intrinsic limitations, imaging at THz frequencies offers a compelling alternative and inherent advantages. For instance, visible and infrared imaging suffer from poor penetration through opaque media, while X‐rays, despite offering perspective imaging, pose significant safety risks due to high‐energy photons causing ionization and molecular damage in biological tissues. Conversely, THz rays are non‐invasive and non‐ionizing [12].

The critical demand for non‐destructive and non‐contact imaging methods, essential for ensuring user safety and preserving delicate objects, is exactly where THz radiation demonstrates its strengths. THz waves can penetrate dielectric materials and can thus be used to detect explosives (for example, C‐4, HMX, RDX, and TNT) and illegal drugs (for example, methamphetamine and heroin), which have characteristic features in the THz range [13]. Beyond security, there is a high demand for THz hyperspectral imaging (HSI) systems (on both centimeter and millimeter scales), for quality assessment or control across many industries (i.e., electronics, wi‐fi communications, pharmaceuticals, maintenance), for medical and biochemical diagnostics, and for the healthcare market (dentistry, dermatology/oncology) [14]. While its relatively long wavelength might seem to limit far‐field spatial resolution, cutting‐edge near‐field imaging approaches have ingeniously overcome this hurdle, enabling THz imaging at the nanoscale [15, 16, 17, 18]. This unique combination of safety, penetrating power, and high‐resolution capabilities positions THz technology as a pivotal tool for advanced analysis and imaging.

Furthermore, the energy scale of THz frequency photons provides direct access to quasiparticle interactions and collective phenomena in materials, such as surface plasmon polaritons [19, 20], enabling applications in spectroscopy, ultrafast dynamics, and coherent control of light‐matter interaction. Notably, THz photons interact strongly with a broad range of quantum and electronic materials, including superconductors [21], topological insulators [22, 23], semiconductors [24], and various 2D systems [25], such as graphene [19], black phosphorus [15], and transition metal dichalcogenides [26].

Despite these clear advantages, THz HSI has still to gain extensive adoption owing to its reliance on time‐domain spectrometers (TDS), based either on costly and cumbersome ultrafast lasers or on low‐efficiency photoconductive antennas [27], both conventionally providing slow (1 kHz scan rates) data acquisition rates, hence limiting market applications.

The advent of grain‐of‐rice‐size THz lasers on a chip, operating at 250 K [28], a temperature reachable with a plug‐in cooler, and of chip‐scale THz frequency combs [10], currently opens concrete perspectives for the development of compact THz HSI systems. At this point, however, the availability of a high‐volume, wafer‐scale, reliable, and low‐cost THz detector production process becomes crucial.

THz focal plane arrays (FPAs), commonly adopted in multipixel imaging systems, are predominantly implemented using either microbolometers [29] or complementary metal‐oxide‐semiconductor (CMOS) image sensors [30]. Commercially available microbolometers have a low NEP (∼30 pW·Hz^−1/2^), and hence are suitable for detecting weak signals [29]. However, a key drawback is their inherently slow response time (τ), which typically ranges from ∼10 to 1000 µs, limiting their effectiveness in applications requiring high temporal resolution.

Conversely, CMOS‐based field‐effect transistors (FETs) provide a compelling alternative due to their much faster response times (<1 µs), suitable for high‐speed imaging [30] and NEPs deeply sensitive to the gate lengths [31].

For instance, when the gate length is reduced to 90 nm, the NEP can be as low as 63 pW·Hz^−1/2^ at a frequency of 2.5 THz. Further reductions in gate length may yield additional sensitivity improvements; however, they also increase fabrication complexity and impose stringent lithographic and variability constraints, particularly in the multi‐THz regime. At these frequencies, system‐level considerations—such as pixel pitch, array scalability, and readout integration—become as critical as ultimate device sensitivity.

In this context, a graphene‐based front‐end offers a complementary technological route. By delegating the THz detection function to graphene devices and co‐integrating them with a CMOS readout integrated circuit (ROIC)—for example, via flip‐chip bonding or heterogeneous integration—the requirements on the CMOS node can be relaxed relative to fully monolithic, nanoscale CMOS detectors. This architectural separation enables greater flexibility in system design, potentially simplifying the ROIC implementation while preserving high‐frequency operation. Importantly, the devices investigated in this work are fully CMOS‐compatible, and their integration can be pursued through monolithic or heterogeneous schemes, providing a scalable and manufacturable pathway toward graphene‐enabled THz focal‐plane arrays.

Here, we propose ultrafast single‐layer graphene (SLG) detectors exploiting the photo‐thermoelectric effect (PTE) as individual pixel elements and devise the first graphene‐based room temperature prototypes of broadband THz frequency cameras. Large‐area (cm^2^) single‐crystal CVD‐grown SLG, with mobility up to µ = 7000 cm^2^V^−1^s^−1^, combined with a planar antenna and on‐chip multiplexing circuitry, are implemented to ensure a combination of high‐speed (sub ns [32], limited by the electron cooling times [33], a feature that is still lacking in alternative technological and commercial platforms for FPA systems) and low NEPs.

Hot‐carrier‐assisted PTE is an extremely efficient detection mechanism in SLG [25, 34, 35, 36]. Effective carrier heating, which stems from the low (1.2 × 10^11^ Jm^−2^K^−1^ at 50 mK [37]) specific heat of SLG's electrons and high SLG optical phonon energy (∼0.2 eV [38]), can result in large electronic temperature (*T*_e_ ∼1000 K) gradients [39], Δ*T*_e_, under photoexcitation, pushing *T*_e_ far above the lattice temperature [40]. The ultrafast electron dynamics [40], combined with the absence of a bandgap, can enable broadband and high‐speed PTE detection. Unlike photovoltaic or bolometric effects [35], PTE can be activated in asymmetric or asymmetrically coupled FETs [36] under zero‐bias operation (i.,e. zero dark current), thus ensuring low power consumption and reduced flicker noise [41].

Recent advancements in scalable SLG production and transfer technologies [42, 43, 44, 45, 46], along with the development of large‐area hBN fabrication protocols [34], have enabled the successful integration of SLG into CMOS platforms to develop broadband sensor arrays operating across a wide spectral range—from the UV region (4–400 nm) to the vitible‐near infrared (900–2000 nm) [47]. CVD SLG has also been integrated with lithium niobate, a thermally polarizable material, for devising mid‐infrared pyroelectric bolometers showing a coefficient of resistance up to 900 % K^−1^ (ref. [48]), making them potential for thermal imaging.

Single‐crystal CVD graphene integrated with photonic waveguides has been used to develop ultrafast, zero‐dark‐current photodetectors for telecom datacom applications [49], and wafer‐scale integration of electro‐absorption modulators incorporating more than ten thousand SLG matrices demonstrates state‐of‐the‐art performance and scalability for graphene‐based hybrid photonics [50]. In parallel, graphene‐ink bolometric detectors implemented in a THz microbolometer array imaging system (MAIS) for biomedical detection have been recently reported [51], with an operational bandwidth of 1–5 THz, average response times of 0.246 s, and peak responsivities of 8.95 × 10^5^ V W^−^^1^. In this approach, each detector pixel consists of a millimeter‐scale standalone chip that is externally assembled and electrically interconnected to form a focal‐plane array spanning several centimeters. While this hybrid assembly strategy effectively enables proof‐of‐concept imaging and high responsivity, it intrinsically limits pixel density, increases system complexity, and poses challenges for scaling toward large‐format, high‐pixel‐count focal‐plane arrays. Moreover, the bolometric operating principle requires continuous electrical biasing, resulting in static power dissipation that may become prohibitive for dense arrays.

Although this approach compellingly demonstrates the potential of graphene‐based THz detection, a complementary technological route based on monolithic, lithographically defined on‐chip integration of SLG‐based THz receivers—operating under zero source–drain bias and with sub‐millimeter pixel pitch—offers a distinct and scalable pathway toward compact, low‐power, and high‐density graphene‐based THz focal‐plane arrays.

It is worth noting that earlier demonstrations of antenna‐coupled CVD graphene detectors at THz frequencies relied either on individual devices [52] or on distributed detector architectures [53], which lacked independent pixel addressability and electrostatic control. As a result, these implementations did not enable pixel‐resolved optimization, tuning, and characterization within a focal‐plane array. In this context, the present work addresses a critical gap by experimentally demonstrating a scalable array of individually gated and electrically addressable graphene THz detector pixels, enabling pixel‐level performance analysis and providing a practical framework for the up‐scaling of graphene‐based THz imaging systems.

## 2 Device Design and Fabrication

In the device architecture exploited in the present work, we developed individual pixels that are THz photodetectors based on *p‐n* junction graphene field‐effect transistors (GFETs) that operate via the PTE effect (Figure 1a). In a PTE‐based device, the generation of photovoltage or photocurrent occurs when a temperature gradient is induced across a junction composed of materials with differing Seebeck coefficients (*S*) [35]. Upon illumination with a THz beam, localized heating at the junction leads to a thermoelectric voltage due to the Seebeck effect. The magnitude of this photovoltage is directly proportional to the product of the difference in Seebeck coefficients across the junction and the induced temperature gradient, Equation (1). This measurable signal serves as a key performance metric for evaluating the detector's sensitivity and responsivity. The induced photovoltage Δ*u*​ can be quantitatively described by the relation:

(1) $$\Delta u=\int_{s}^{d}\frac{\partial T_{e}}{\partial x}\cdot S\left(x\right)\mathrm{dx}=\Delta T_{e}\cdot \left(S_{2}-S_{1}\right)=\Delta T_{e}\Delta S,$$

where *S*_1_ and *S*_2_ are the Seebeck coefficients of graphene on the two sides of the junction, Δ*T_e_* is the temperature difference of carriers at the center of the antenna – junction with respect to the carriers on the leads, and Δ*S* is the difference between *S*_2_ and *S*_1_. According to Equation (1), the optical response is proportional to Δ*S* and Δ*T_e_*. Here, Δ*S* is related to the properties of graphene itself, whereas Δ*T_e_* is affected by the intensity of the incident electric field and by the heat sink temperature. More details are provided in the Supporting Information.

**FIGURE 1:**
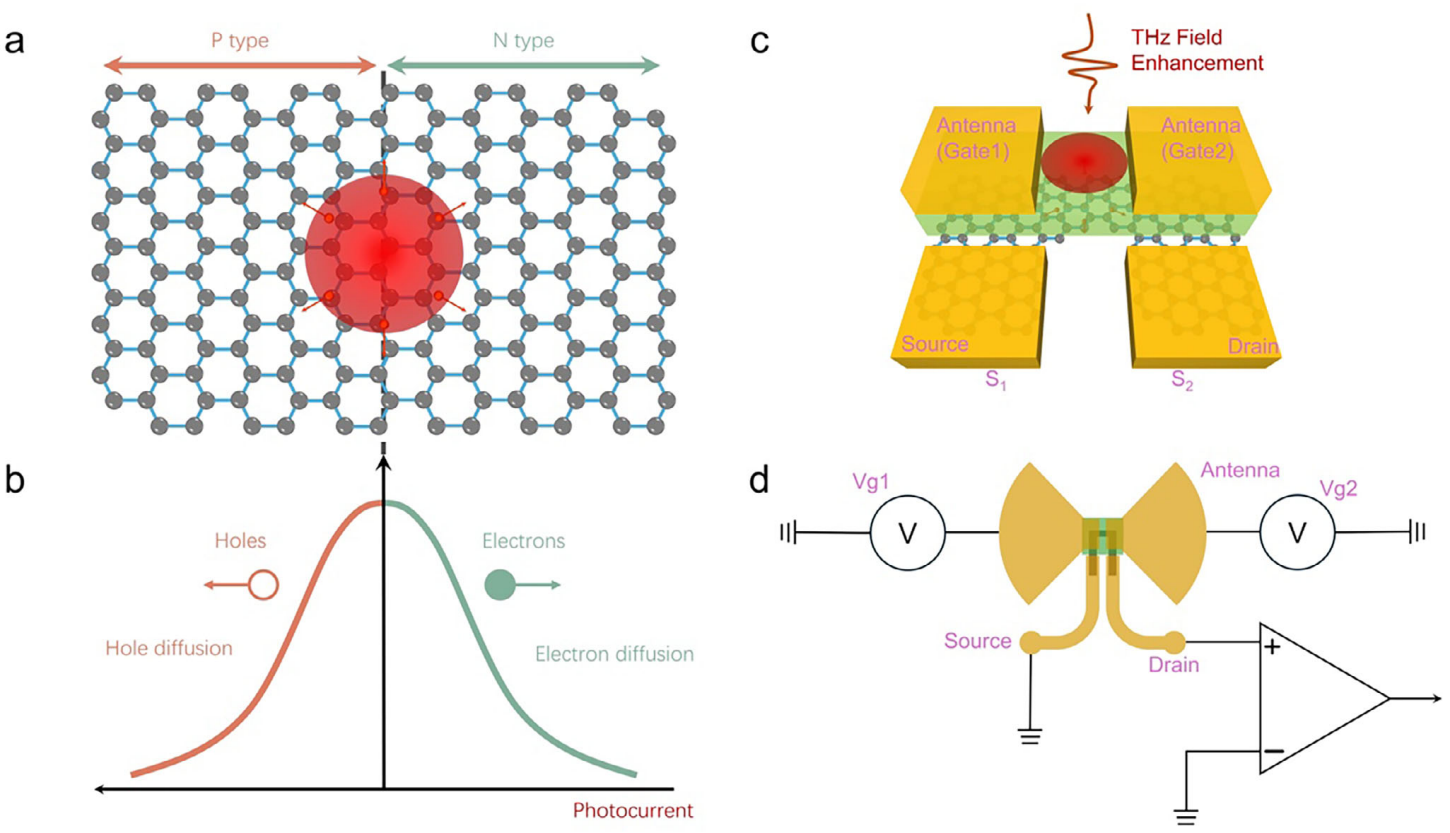
Individual pixel detection principle. (a) Photocurrent generation in a p‐n junction graphene detector. (b) The sub‐picosecond oscillation of THz waves exceeds the electron momentum scattering period (10–100 fs) in graphene, leading to diffusive transport where electron motion is incoherent with the driving electric field. Hot carriers diffuse away from the p‐n junction (hot) region. (c) PTE based p–n junction graphene THz detector architecture. The photocurrent or photovoltage arises at the interface between regions of different *S* (*S*_1_ and *S*_2_) via localized heating (Δ*T*_e_, red area) induced by the near‐field of the antenna, excited by the THz beam. This interface is defined by local gating (*V*_g1_ and *V*_g2_) that induces spatially controlled doping. (d) Circuitry of the PTE THz detector. The antenna lobes act as tunable gates. The photon‐generated voltage at the *d* electrode is amplified by the preamplifier and read by a lock‐in amplifier.

Due to its distinctive linear band dispersion and zero bandgap, SLG exhibits a conical intersection of the conduction and valence bands at the Dirac point. Under varying gate voltages, the dominant charge carriers within graphene can be either holes, electrons, or a mixture of both, revealing its ambipolar transport characteristics (Figure 1b). The photo‐response of SLG‐based PTE THz detectors may exhibit a dependence on carrier mobility [35], which governs both the magnitude of the electronic temperature gradient via enhanced thermal diffusion and the energy sensitivity of the local *S*, as shown in Figure 1a,b [54]. Hexagonal boron nitride/graphene/hexagonal boron nitride (hBN/graphene/hBN) heterostructures assembled via mechanical exfoliation can allow reaching low noise equivalent powers ∼10 pW Hz^−1/2^ in GFETs at room temperature [55]. However, the channel areas devised so far with exfoliated layered material heterostructures are still rather small (<mm [2]) due to the difficulties in achieving performance reproducibility across large wafers.

In our device implementation, we employ a large‐area SLG synthesized by chemical vapor deposition (CVD) and covered by a thin oxide (HfO_2_). Although CVD‐grown graphene inevitably contains structural defects and exhibits lower carrier mobility (µ = 1000–7000 cm^2^V^−1^s^−1^) [56, 57] than exfoliated graphene, it still enables high‐sensitivity photodetection (noise equivalent powers NEP∼ nW/Hz^1/2^) [34, 36], while being inherently scalable. Moreover, SLG is compatible with the highly mature silicon‐based platform, allowing its low‐cost and large‐scale integration into optoelectronic networks and multipixel CMOS read‐out integrated circuits. Engineering a high‐sensitivity graphene PTE detector requires both effective modulation of carrier density and efficient coupling of the incident THz beam into the active region.

To achieve this, each detector pixel is equipped with a 0.8 µm wide graphene channel, where a bow‐tie‐shaped broadband antenna, having a bow radius of 24 um, matched with a ∼2.9 THz central frequency, acts as a dual‐purpose gate electrode—enabling electrostatic tuning of the carrier distribution while simultaneously coupling the THz field onto the GFET channel. We note that bow‐tie antennas typically cover bandwidths >30 % with respect to the central frequency [52, 58], the proposed THz devices have a frequency coverage >0.8 THz. In the context of the present work, the term “broadband” refers to the antenna‐coupled device architecture, since bow‐tie plasmonic antennas are known to provide wide THz coupling bandwidths and can be lithographically redesigned to target different frequency bands, rather than to a frequency‐resolved responsivity measurement performed within this study. Drain (d) and source (s) electrodes are connected to the channel to extract the photovoltage and act as ground, respectively. The scheme of the detector is shown in Figure 1c,d.

For the implementation of THz cameras, we adopt two distinct design strategies (Figure 2). In a first scheme, we design and engineer a 2D detector array, in which each detector unit is individually addressable and with an independent read‐out (Figure 2a). In the second case, we engineer a linear THz camera architecture, where pixel elements are arranged into a single row with shared source‐ground lines and partially shared gate (*g*) electrodes (Figure 2b). This configuration significantly reduces the number of electrodes and simplifies the overall device architecture, offering a compact and scalable solution for line‐scan THz imaging.

**FIGURE 2:**
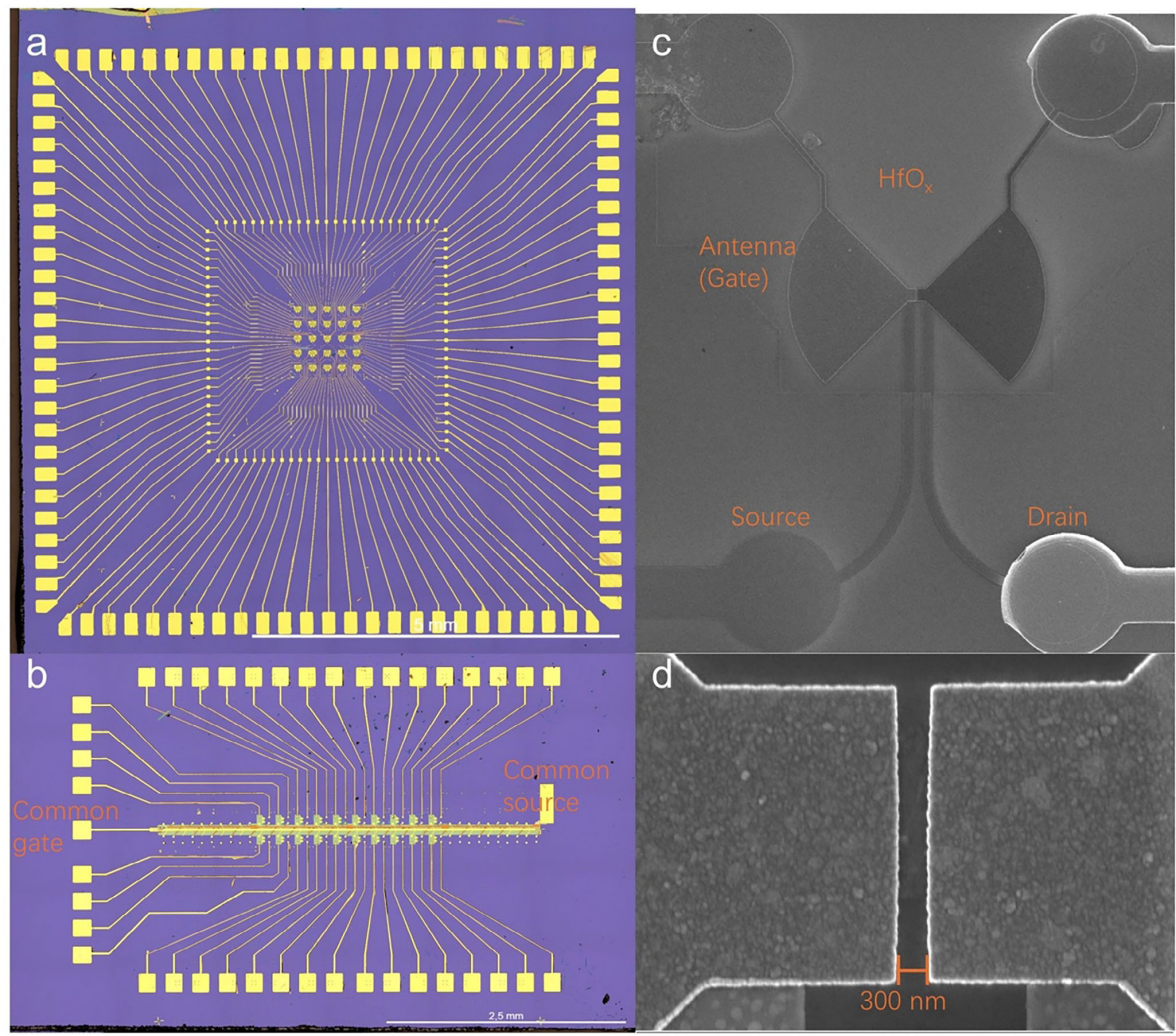
Optical images of the two multipixel terahertz arrays embedded in the devised THz cameras. (a) A 5 × 5 square array camera. The period is 200 µm. Each detector has four electrodes with two tunable gates, a grounded source, and a drain. (b) Linear array with 20 pixels. The *x* and *y* pixel distance is 200 µm. The detectors share a common source electrode and a common gate. Each detector has an individual gate and drain. (c) SEM image of a single pixel (d) SEM image of the antenna slit having a 300 nm gap.

## 3 Results

### 3.1 Characterization of the Devices

Individual GFETs are fabricated using a combination of electron beam lithography (EBL, Zeiss UltraPlus), atomic layer deposition (ALD, Oxford OpAL), and metal evaporation on a high resistivity SiO_2_/Si substrate (*ρ* >20 kΩ·cm). The p‐n junction geometry is defined by a couple of top split‐gates over an H‐shaped channel, created through reactive ion etching (CF_4_/O_2_ mixture, 25 W, etch rate 3 nm s^−1^). The *s* and *d* electrodes are lithographically defined along the SLG channel at a relative distance *L*_c_ = 4 µm. The channel width *W*_c_ = 0.8 µm. The fabrication process is then completed by depositing 5/40 nm Cr/Au through thermal evaporation and liftoff. This provides electrical contact to the SLG channel, with a contact resistance (*R*_0_) of about *R*_0_≈10 kΩ. A thin oxide layer (HfO_2_ ≈30 nm thick) is then grown at 130°C via atomic layer deposition (ALD) on the channels as the top‐gate oxide. A set of two top‐gate electrodes, with a gap separation ≈300 nm (Figure 2c,d), is then lithographically defined and metallized with a 5/90 nm Cr/Au layer. In the p‐n junction geometry, the asymmetry required for PTE generation is determined by the longitudinal variation of the SLG Seebeck coefficient. The gate electrodes are designed in the shape of two halves of a bow‐tie antenna, having a flare angle of 90°. At 2.86 THz and for normal incidence, the selected geometry leads to a field enhancement ≈40 between the antenna branches, when THz waves are impinging on the chip with a polarization parallel to the antenna axis from the air side. The silicon chip is finally mounted on a double‐sided printed circuit board (PCB) with multi‐channel leads. Both sides of the PCB are designed to accommodate signal readout from imaging chips: one side features a 16 × 4‐channel layout for 16‐pixel chips, while the other side provides a 32 × 4‐channel layout for 32‐pixel chips, enabling compatibility and scalability.

In a THz camera, as the number of integrated pixels increases, the complexity of the required control circuitry grows accordingly. However, if all pixels exhibit uniform and predictable device characteristics, the control architecture can be significantly simplified. Given that the electrode and antenna designs are identical across all detector units, the uniformity of the array is predominantly determined by the consistency of the electrical performances retrieved in the GFET channels, which is, in turn, deeply affected by the uniformity of the CVD‐transferred graphene itself. To assess this, we first employ micro‐Raman spectroscopy to statistically evaluate the spectral properties of graphene across different regions of the large‐area SLG. A representative Raman spectrum is shown in Figure 3a. We found peaks at Pos (G) = 1590 cm^−1^ and Pos (2D) = 2678 cm^−1^. The 2D and G peaks are single Lorentzian with full‐width at‐half‐maximum FWHM (2D) = 52.5 cm^−1^, and FWHM (G) = 20.7 cm^−1^. The 2D to G peak intensity and area ratios are I(2D)/I(G) = 1.07 and A(2D)/A(G) = 2.01, corresponding to a Fermi energy of ∼25 meV [59]. I(D)/I(G) = 0.245, indicating the presence of Raman active defects.

**FIGURE 3:**
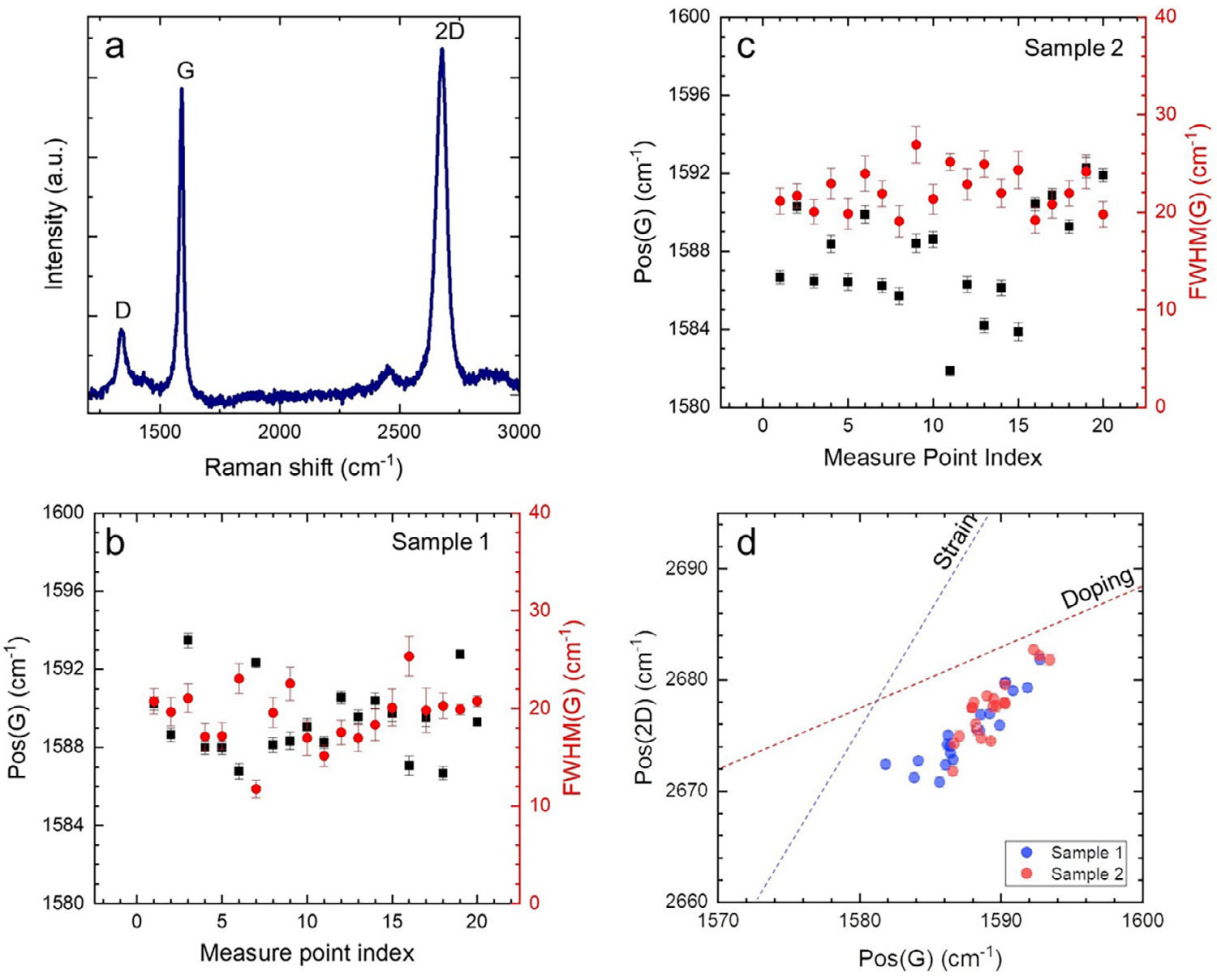
Raman characterization. (a) representative Raman spectrum. (b,c) Pos(G), Pos(2D), and their FWHM were measured on 20 points for (b) samples 1 and (c) samples 2. Pos(2D) vs. Pos(G) frequency extracted from Raman spectra acquired in different positions on the sample. (d) The dispersion of the data points along the doping axis indicates a large variability in doping for both samples and the presence of tensile strain. Data are presented as mean values + standard error of the mean bar, calculated following the relative error method, namely considering the standard error of Pos(G), Pos(2D), and their FWHM extracted from Raman spectra.

The statistical pixel analysis (Figure 3b,c) shows that the position of the G peak (Pos(G)) for the transferred CVD graphene ranges from 1582 to 1589 cm^−1^ and the FWHM ranges from 11.8 to 26.9 cm^−1^. From the analysis of the Raman spectra, we note that transferred graphene displays a p‐type doping and a corresponding hole concentration of ≈4.7 × 10^10^ cm^−2^ [60], in agreement with typical observations of CVD‐grown graphene transferred on SiO_2_ [61]. Importantly, we note that this initial doping is typically affected by the nano‐fabrication procedure. Specifically, HfO_2_ deposited by ALD has been observed to impact the carrier concentration of graphene, shifting it toward n‐doping [62].

The analysis of the 2D‐peak vs. G‐peak frequency in different positions on the sample (Figure 3d) indicates variable doping and the presence of compressive (Figure 3d) strain for both samples.

Minimizing strain through the optimization of transfer methods not only enhances the structural integrity of graphene but also leads to more consistent and uniform electronic properties [63]. Indeed, graphene is highly sensitive to strain, and any induced strain can lead to unpredictable localized deformations. Therefore, smoother SLG layers show lower residual doping levels, since strain often induces carrier doping as a consequence of lattice distortions that affect the electronic structure of graphene.

To evaluate the device‐level performance, we individually characterized the electrical behaviors pixel‐by‐pixel (see Supporting Information) and investigated the gate voltage dependence of the resistance curve to extract R_0_, mobility, and residual carrier density (n_0_). To this end, we opted for sweeping the bias of one gate (left, V_GL_) with respect to the other (right, V_GR_), which was conversely kept equal to zero. This strategy allows us to optimize the device optical performance near the maximum responsivity point (see Supporting Information), without requiring a too complicated biasing scheme to activate the p‐n junction PTE response.

This single‐gate biasing approach was intentionally adopted as a system‐level design choice aimed at simplifying the array architecture and reducing the number of independent control lines, which is a key requirement for scalable focal‐plane arrays. Importantly, previous studies have shown that operating graphene PTE detectors under single‐gate control results in only a modest reduction in responsivity (≈10 %) compared to fully optimized dual‐gate configurations, while preserving the same underlying photo‐thermoelectric mechanism [52]. Therefore, the present biasing strategy represents a favorable trade‐off between peak device performance and architectural simplicity for large‐scale integration.

The analysis shows that the fabrication yields of the two cameras reach 92 % and 80 %, respectively, indicating high uniformity and robustness of our process. Despite some variation in the resistance values of the graphene channels, nearly all detectors within each camera exhibit consistent behavior, with only minor discrepancies (see Supporting Information). For the linear‐array camera, all pixels demonstrate good responsivity at a gate voltage of 1 V, enabling the use of a single power supply to drive the gate electrodes uniformly. Similarly, for the square‐array camera, a gate voltage of 0.5 V ensures uniformly high (up to 40 V/W) maximum responsivities across all pixels, providing a reliable basis for collective gate control.

We then tested the devices optically. We adopt a single‐mode 2.86 THz quantum cascade laser (QCL) source, delivering 500 µW power to characterize the photo‐response of each pixel element as shown in Figure 4a. The QCL is mounted in a Stirling cooler, and the temperature is kept fixed at 26 K. The QCL beam is then focused by two tsurupica (TPX) lenses onto a circular spot with radius ≈200 µm. The corresponding average intensity at the focal point is *I* = 0.5 W cm^−2^. A square‐wave envelope with frequency *f*_mod_ = 1.333 kHz is used as a lock‐in reference and as a triggering signal for the QCL pulse trains.

**FIGURE 4:**
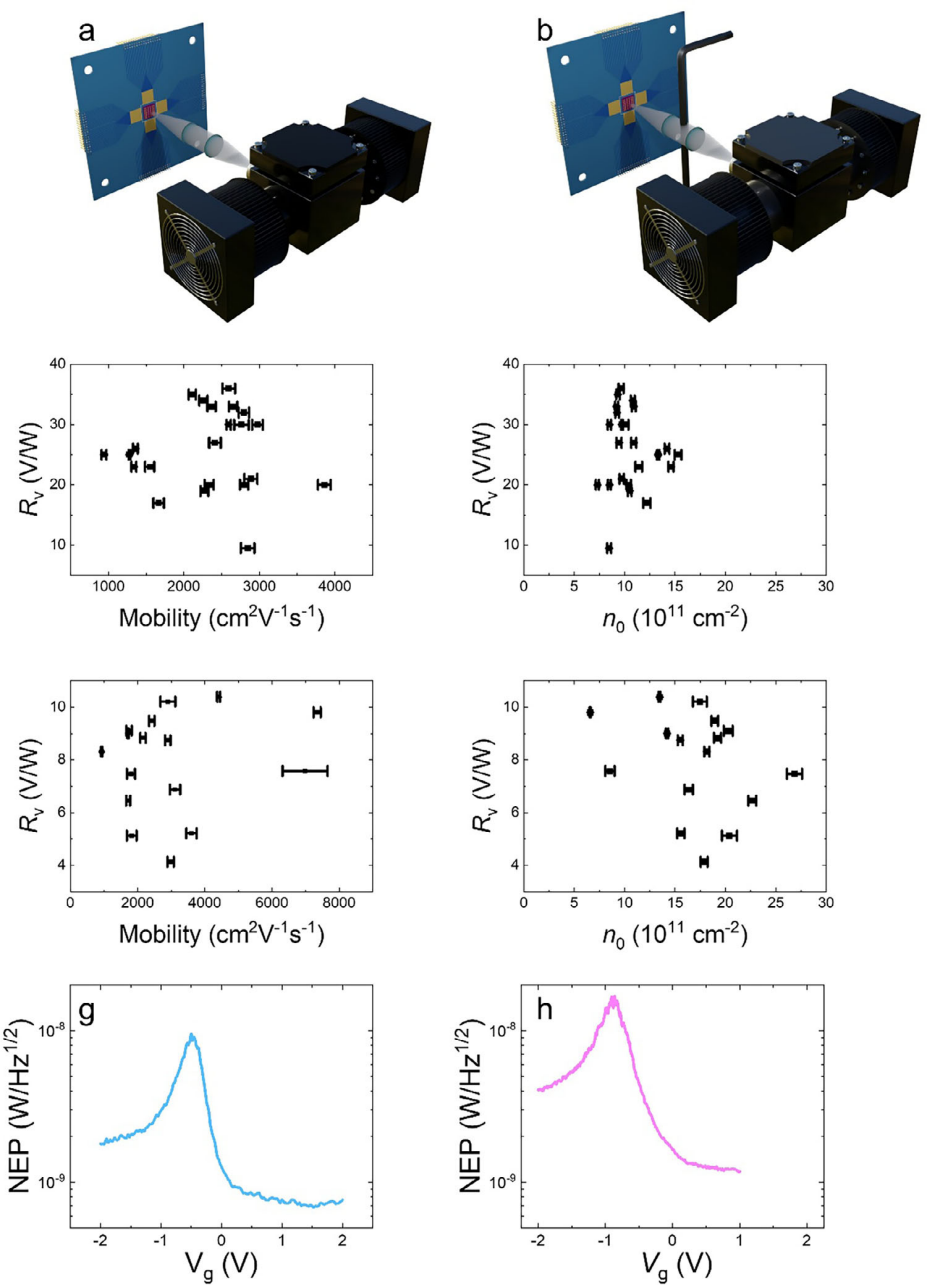
Optical characterization of individual pixels. (a) Schematic configuration of the optical setup comprising a Stirling cryocooler, two lenses, and the array element. Optical measurement setup configuration. The QCL is mounted inside the cryostat and cooled down to 26 K; the temperature was kept fixed at 36 K when the output power is 500 µW. (b) Imaging scheme of a hex key. The hex key is placed between the TPX lens and the beam is focused by two TPX lenses onto the camera. (c,d) *R*_v_ vs. mobility and residual carrier density of individual pixels in the square array camera. (e,f) *R*_v_ vs. mobility and residual carrier density of individual pixels in the linear array camera. (g) NEP vs. *V*_g_ of a typical pixel in the square array camera. (h) NEP vs. *V*_g_ of a typical pixel in the linear array camera.

To extrapolate the responsivity, R_v_, we extract the photovoltage (Δu) at the drain electrode while keeping the source grounded. A voltage preamplifier (DL Instruments, M1201, gain 𝛾 = 1000) allows for amplifying Δu, which is then sent to a lock‐in (Stanford Research, 5210). Δu is then retrieved from the demodulated lock‐in signal (V_LI_) as Δu = $\left(\pi \times 2\sqrt{2}\right)V_{LI}/\gamma$, where the pre‐factor $\pi \times 2\sqrt{2}$ takes into account that the lock‐in measures the RMS of the fundamental Fourier component of the square wave produced by the QCL modulation. *R*_v_ is then calculated from the ratio between Δu and the power impinging on the detector, *P*_a_. Taking into account that the total detector area is smaller than the diffraction‐limited one (S_λ_), the active area is taken equal to A_eff_ = S_λ_ = 𝜆^2^/4 = 2800 µm2, where 𝜆 is the free‐space wavelength [64]. This choice provides a conservative and physically meaningful normalization for free‐space THz illumination, avoiding artificial overestimation of responsivity that would arise from using the much smaller physical detector area. The power impinging on the device P_a_ is evaluated as P_a_ = P_t_·(S_λ_/ S_t_), where P_t_ is the laser power, and S_t_ is the beam spot area.

The individual pixel responsivities are shown in Figure 4c–f for the two sets of cameras. The responsivities vary in the range 25–36 V/W for >86 % of the devices of the square array camera (in Figure 4c,d) and from 4.1 to 10.4 in the linear array camera (in Figure 4e,f), showing a 26 % and 24 % relative standard deviation, respectively. The same variability change is retrieved from the transconductances extracted from the transport data (see Supporting Information). This is a consequence of the residual carrier concentration, which plays a critical role in the photo‐thermoelectric (PTE) response. The PTE responsivity is maximized when the Fermi level is close to the charge neutrality point, where the Seebeck coefficient exhibits a strong gradient with carrier density. In the linear array, the adopted graphene shows higher residual carrier concentration and unintentional doping, resulting in a reduced Seebeck coefficient difference between the p–n junction regions. The performance non‐uniformity observed in the graphene PTE detector array can be attributed to the intrinsic inhomogeneity of the transferred graphene film. Due to the nonuniform spatial distribution of grain boundaries within the graphene layer, local variations in carrier density, mobility, and Seebeck coefficient are introduced. Since the photo‐thermoelectric (PTE) response is highly sensitive to the local Seebeck coefficient and its spatial gradient, these variations lead to pixel‐to‐pixel differences in the generated photovoltage and responsivity. As a result, even under identical device geometry and fabrication batch, the PTE detector array exhibits a certain degree of performance variability.

The correlation between photoresponsivity and residual carrier density for each pixel is summarized in Figure 4d,f, respectively, considering the set of data points extracted from the transport characteristics. We attribute the lower responsivity of the linear array to the larger values of residual carrier density (Figure 4d,f). This correlation stems from the fact that SLG with lower n_0_ is expected to have larger Seebeck coefficients, as predicted by the Boltzmann theory [65].

The combined electrical and optical measurements reveal a high degree of uniformity across the individual arrays, providing a solid foundation for the development of streamlined control schemes in large‐scale terahertz camera systems. We evaluated the NEP as the ratio between noise spectral density (NSD) and responsivity. Here, we assume the NSD to be dominated by thermal fluctuations in the graphene channel, which result in a voltage noise figure given by the Johnson–Nyquist formula: NEP = (4*k*_B_ *RT*)^1/2^/*R*_v_, where *k*_B_​ is the Boltzmann constant, *T* is the temperature, *R* is the device resistance, and *R*_v_​ is the voltage responsivity. This method results in a gate‐dependent NEP, as displayed in the two representative graphs in Figure 4g,h. For the square array camera, its best NEP is lower than 1 nW/Hz^1/2^, while the best NEP is <2 nW/Hz^1/2^. It is worth mentioning that the individual elements are expected to show response times of the order of ∼1 ns [34]. The setup‐limited detection speed, measured at the single pixel level (see Supporting Information), is 2.6 MHz, corresponding to a response time of ∼60 ns.

### 3.2 Readout of the Cameras

To enable imaging, a time‐division multiplexing approach has been employed to sequentially read out the signal from each pixel in the camera.

Since the optical response of individual pixels has been pre‐characterized, image reconstruction is performed using a normalization scheme rather than relying on absolute photo‐response values, namely:

(2) $$R_{\mathrm{Cam}}=\frac{R_{\mathrm{sign}}-R_{\mathrm{noise}}}{R_{v}}$$

where *R*_Cam_ is the normalized pixel response, *R*_sign_ is the readout signal of the pixel, *R*_noise_ is the noise level at the end of the amplification chain. To be consistent, we use the normalized signals since even if the individual pixel responsivity shows good uniformity, the overall response still differs slightly.

For the linear array camera, due to the different imaging ranges along the two spatial dimensions, line scanning is required during imaging. This is achieved by translating the camera along the direction orthogonal to the array line, that is, along the vertical direction. To enable multiplexed readout, a signal switching module (Arduino UNO3) and a multiplexer module (Texas Instruments, V599 Multiplexer) are implemented. The signal switching module controls the channel selection of the multiplexer, which in turn handles both the acquisition and readout of the camera signals, ultimately enabling image formation, as illustrated in Figure 5. The switching module is realized using a custom‐designed circuit board that provides 14 control lines for selecting signal pathways within the multiplexer. The multiplexer module includes 16 input channels (C0–C15), a single output (SIG), four digital control lines (S0–S3), a supply voltage (Vcc), and an enable signal (EN). By configuring the logic levels of S0 through S3, any input channel can be routed to the output. For example, setting S0 = 0, S1 = 0, S2 = 0, and S3 = 0 results in channel C(2^*S*0 + *S*1 + *S*2 + *S*3^ − 1) = C0 being selected. For the 16‐pixel THz cameras, a single multiplexer module is sufficient. For larger arrays, multiple multiplexer modules can be combined and time‐multiplexed via their respective EN signals, enabling scalable signal acquisition for high‐pixel‐count terahertz imaging systems.

**FIGURE 5:**
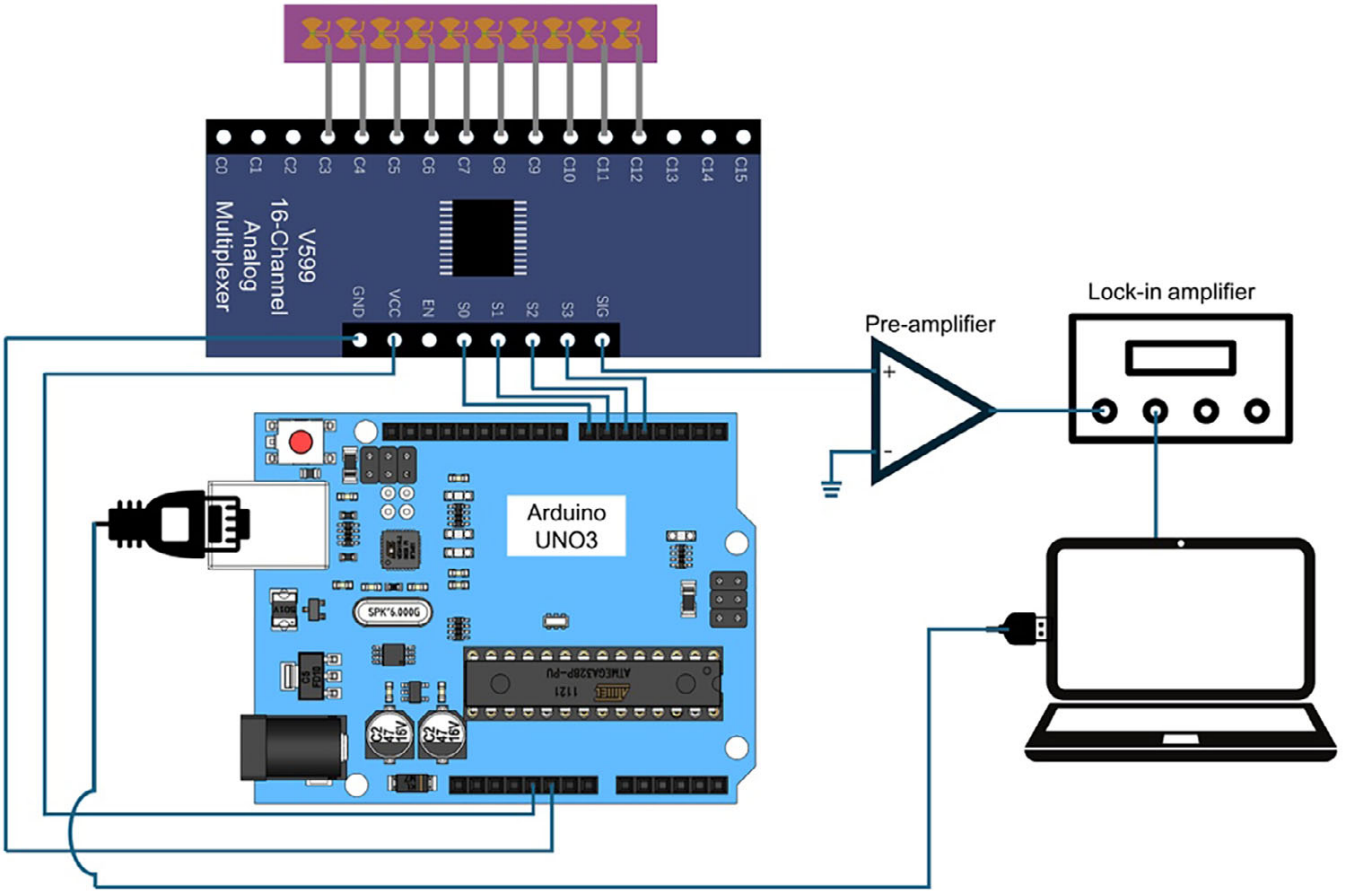
Readout schematics. In the readout system, the photodetector's photo‐response is routed to the output port of the V599 multiplexer board from C0 to C15, with multiple switches embedded between the input and output channels. The switching logic is governed by voltage levels applied to digital control lines S0–S3. An Arduino UNO board configures these control lines and simultaneously powers the V599 board, eliminating the need for an external power supply. The final signal is delivered via the SIG output of the V599 board, subsequently amplified by a preamplifier and a lock‐in amplifier before being digitized and recorded by a computer.

The response time of a typical detector in the array camera is 60±1 ns, indicating a 2.6 MHz bandwidth. However, limited by the speed of the readout electronics, the response time of the system is 1 s. Therefore, in order to enable real‐time and fast acquisition of images, a high‐speed readout circuit needs to be developed in the future.

### 3.3 Imaging Results

To validate the performance of the developed THz camera, we first employed the QCL beam itself as the imaging target. Owing to its pronounced spatial variation in intensity across the focal (*x‐y*) plane, the QCL beam serves as a natural probe for evaluating the spatial imaging performance of the camera. Figure 6 displays the bi‐dimensional (2D) focused beam image acquired with the linear array THz camera using the upper and lower linear matrices, respectively. Given that the QCL beam has a radius of ∼200 µm and the pixel pitch of the detector array is 200 µm, the entire beam profile can be effectively captured using only five adjacent pixels. In this scan, each pixel was individually biased at its optimal gate voltage (V_Gn_), rather than using a global gate control. However, the optimal gate bias for maximum photo‐response differs slightly between pixels, which complicates simultaneous multi‐pixel readout. To achieve the images shown in Figure 6, we therefore adopted a point‐by‐point scanning method in which each pixel was biased at its individual optimal V_Gn_ during measurement. During each step, the output signals from different pixels were sequentially read. The scan was performed with a 30 µm step size over a 1200 µm range along the *y*‐axis, fully encompassing the QCL beam profile. By translating the camera along the *y*‐axis, and sequentially reading out each pixel, we reconstructed the spatial intensity profile of the QCL beam based on the detector outputs. Notably, different combinations of pixels can be used to reconstruct the beam, offering flexibility in imaging strategies (see Figure 6). This approach yields a high‐fidelity reconstruction of the beam profile and serves as a reliable benchmark for evaluating the accuracy of the proposed imaging modality, even considering that simultaneous different gate biasing conditions are required.

**FIGURE 6:**
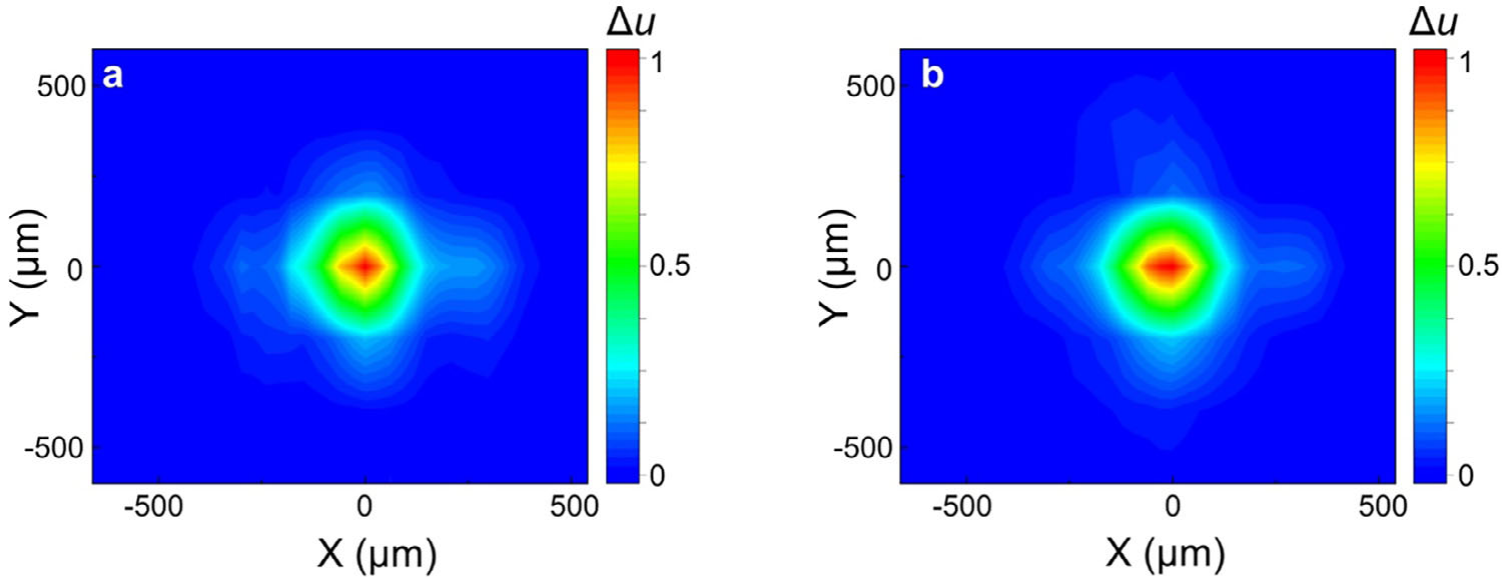
Scanning of the QCL beam with individual gate control. Pixel to pixel 2D scanning with (a) the upper linear array camera and (b) the lower linear array camera of the intensity distribution of the focused THz QCL beam. For both images, the THz beam is reconstructed using a linear scanning approach. The beam is first focused onto the central pixel of a linear THz camera. Each pixel is individually biased to its optimal gate voltage—determined from prior single‐pixel photo‐response measurements—to maximize sensitivity. The corresponding output signals are then recorded. The camera is subsequently translated in 30 µm steps along the *y*‐axis, with signals read at each position under optimal biasing conditions. This process is repeated until all detector outputs reach the noise level (the THz beam falls outside the array).

To address the challenge of bias nonuniformity, we systematically analysed the photo‐response–gate voltage characteristics across the pixel array. Owing to the highly consistent response behaviour among pixels (see Supporting Information), a global V_G_ can be applied with only modest signal degradation.

After normalization to compensate for possible sensitivity variations, the reconstructed beam profile remains quantitatively the same. This strategy significantly improves imaging efficiency and provides a scalable pathway for future readout architectures in large‐format 2D‐material‐based THz cameras. In our current implementation, all pixels are uniformly biased at +1 V. The reconstructed beam profile using this fixed‐bias mode is shown in Figure 7b.

**FIGURE 7:**
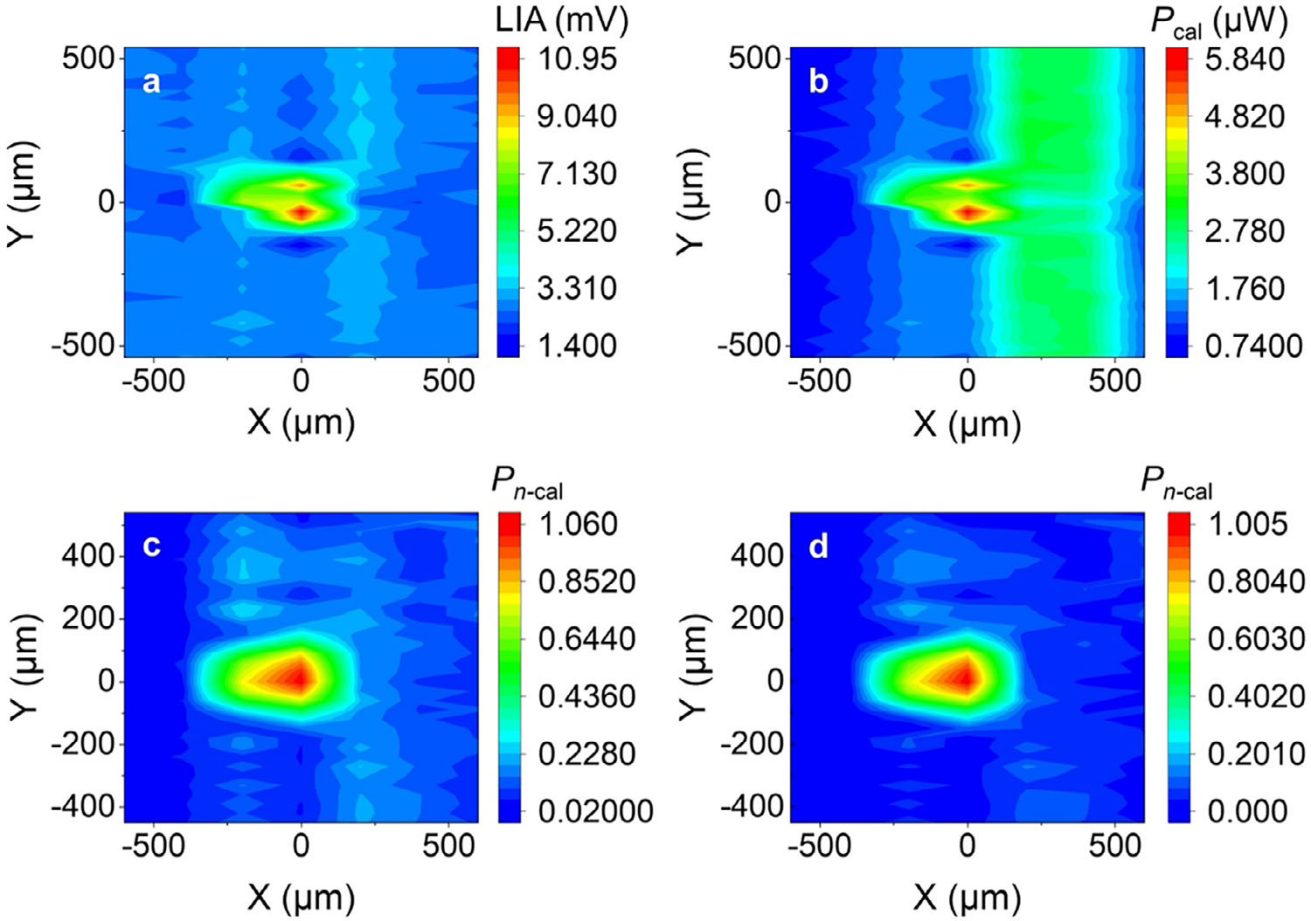
Imaging reconstruction procedure with uniform gate bias. (a) Beam reconstruction using raw, uncalibrated data directly from the lock‐in amplifier output. (b) Reconstruction based on a, with pixel signals normalized by their responsivity at a gate voltage of 1 V. (c) Building on (b), each pixel underwent a dark‐field measurement without THz illumination to quantify its noise level, which was subsequently subtracted during image reconstruction. (d) As the noise level is not temporally stable, long‐term measurements were performed to determine the average noise baseline. The resulting beam image incorporates optimized noise calibration for improved fidelity.

Therefore, our calibration protocol, described by Equation (2), involves a two‐step procedure. First, the THz beam is blocked to obtain the baseline noise response of each pixel in the absence of THz illumination. This noise baseline is then used for correction during the actual imaging under exposure at the THz beam, effectively introducing a calibration step prior to the measurement. Based on this protocol, we selected one representative linear pixel combination from the THz camera array to reconstruct the QCL beam, applying both normalization and noise‐level correction, as shown in Figure 7c,d. In Figure 7c, the image reconstructed after initial normalization and baseline correction still exhibits residual noise. We then include a modification to the noise level evaluation that combines high‐sensitivity (1 mV) and long integration time (1 s) for the lock‐in instrument. We note that the long lock‐in integration time is adopted to suppress pixel‐to‐pixel switching noise during sequential readout and does not reflect the intrinsic detector speed. The resulting beam image incorporates optimized noise calibration for improved fidelity (Figure 7d). This yields a cleaner beam profile that closely matches the result obtained by point‐by‐point scanning, thereby validating the efficacy of the proposed correction methodology.

To show a concrete application, we then employed the linear‐array THz camera to image a real‐world object. The optical setup is illustrated in Figure 4b. Due to the limitations of the focusing configuration, the THz beam has a relatively small cross‐sectional area (full width half maximum, FWHM ∼250 µm), which prevents the object from being placed between the lenses and the detector array, as it would otherwise block the beam. To validate the spatial tracking capability of the system, we first translated the THz camera along the *x*‐axis. As shown in the video provided in the Supporting Information, the THz spot shifts together with the pixel motion and in the same direction, confirming the spatial fidelity and operational integrity of the imaging system. Subsequently, we placed a hex key between the two collimating lenses to investigate its influence on the THz beam profile, shown in Figure 8a. The resulting intensity profiles are presented in Figure 8b,c. The presence of the hex key clearly distorts the beam shape, in a way visibly consistent with the object's physical placement in the beam path. It should be noted, however, that the reconstructed image does not represent the true scale of the hex key due to the magnification introduced by the optical elements. Nevertheless, the reconstructed profile retains sufficient spatial fidelity to reveal the object's contours. In addition to single‐line array operation, the dual‐line configuration of our THz camera allows for an enhanced imaging strategy. Specifically, we implement a two‐line scanning method, which is faster than single‐line scanning. Given the finite beam width, we demonstrate this method using two scanning cycles, with the results shown in Figure 8c.

**FIGURE 8:**
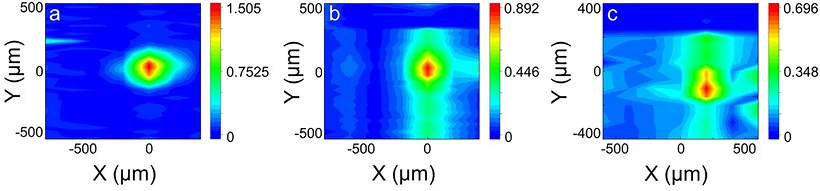
Imaging of a deflected beam. (a) Beam reconstruction without the hex key, using a single linear array camera. (b) Reconstructed image with the hex key inserted, using a single linear‐array camera. (c) Beam reconstruction under the same conditions as in (c) but using a dual linear array camera.

A comparison with Figure 8c reveals that both scanning strategies produce comparable beam profiles, but the dual‐line method achieves this with higher acquisition efficiency. This concept points toward a promising route for further improvements: by constructing THz cameras with 2D or multi‐line array architectures, the imaging throughput could be significantly enhanced.

Apart from linear THz cameras, we also put forward the square array THz camera scheme, as shown in Figure 2a. The imaging system is identical to the linear array camera. Since the squared array camera chip is wider than the beam itself, the entire beam profile can be captured without mechanical scanning. Using the same readout and calibration procedures as for the linear array camera, the imaging results are shown in Figure 9a,b. The reconstructed beam profile closely resembles that obtained with the linear‐array camera, though with reduced resolution along the *y*‐axis. This is due to the fixed pixel spacing in the array, which limits spatial resolution in the absence of scanning.

**FIGURE 9:**
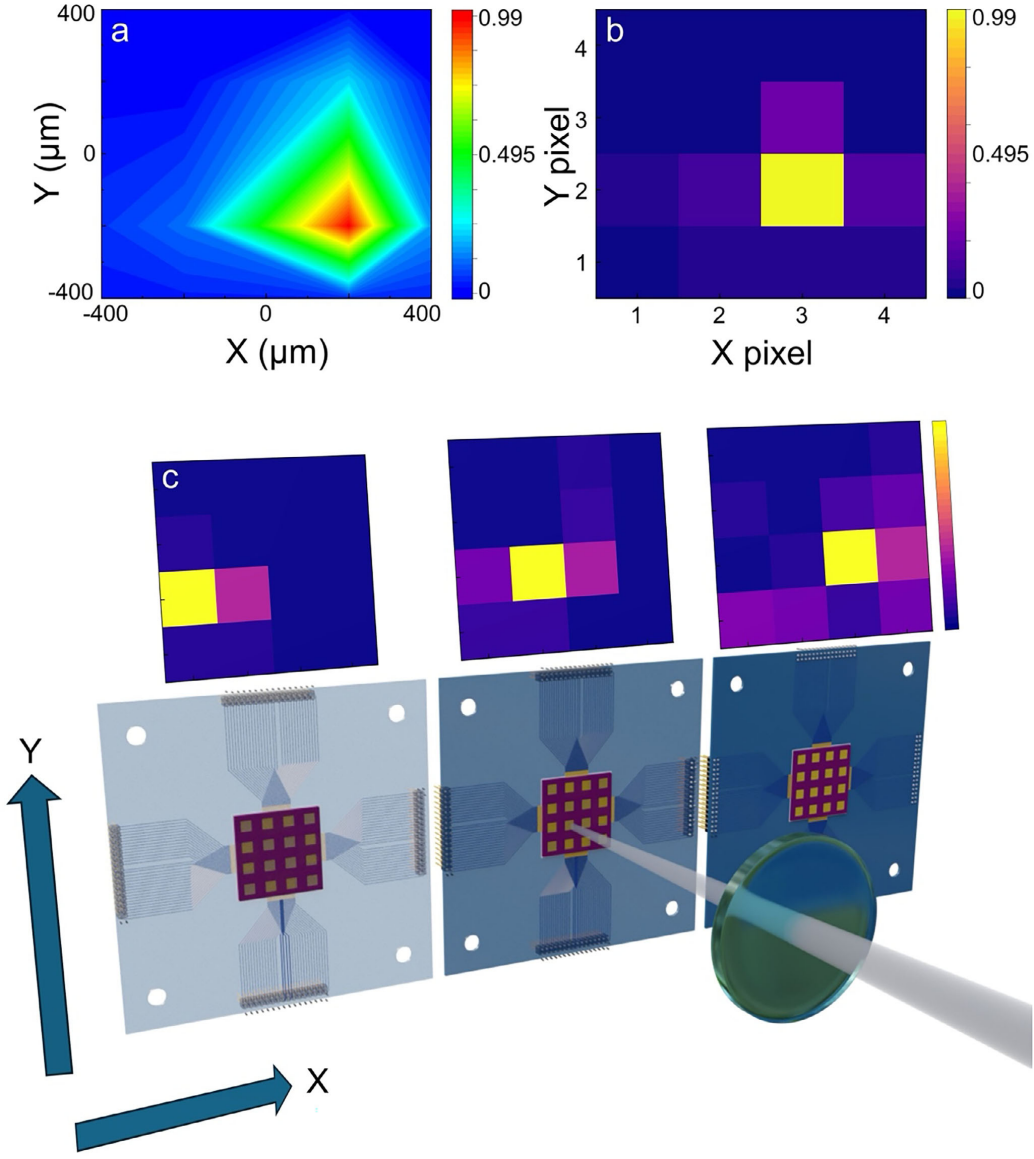
Mapping of the THz QCL beam with the square array. (a) Reconstructed THz beam image acquired with the square array camera, using the same optical configuration and calibration procedure adopted for the linear array camera. (b) Corresponding heatmap of the result in (a), showing the spatial response mapped onto individual detector pixels. (c) When the camera is translated along the X direction, as the X arrow shows, the THz beam sequentially activates pixels along the scan path. A similar response is observed for both the *x* and *y* directions (see Supporting Information).

To illustrate the imaging capability, we present an intensity map in Figure 9b, where the THz beam spot is centered on one of the pixels. Given the relative dimensions of the beam spot size (FWHM ∼200 µm) and pixel pitch (200 µm), the pixels at the beam center appear “lit,” with nearby pixels partially activated, while those farther away remain “dark.” In Figure 9c, we investigate how the square camera tracks the THz beam as it is scanned across different pixels. Subsequent snapshots acquired with the square camera reveal how the beam is scanned over various positions on the array.

Finally, to mitigate the limited pixel count of the 5 × 5 array and to better visualize spatial features, we reconstruct a composite image by translating the square‐array camera over a 3 × 3 grid of positions and combining the resulting measurements into an effective 15 × 15‐pixel map (Figure 10). While this composite reconstruction does not represent real‐time focal‐plane imaging, it provides a clearer visualization of the spatial response of the array and demonstrates pixel‐to‐pixel uniformity and spatial consistency across the detector matrix.

**FIGURE 10:**
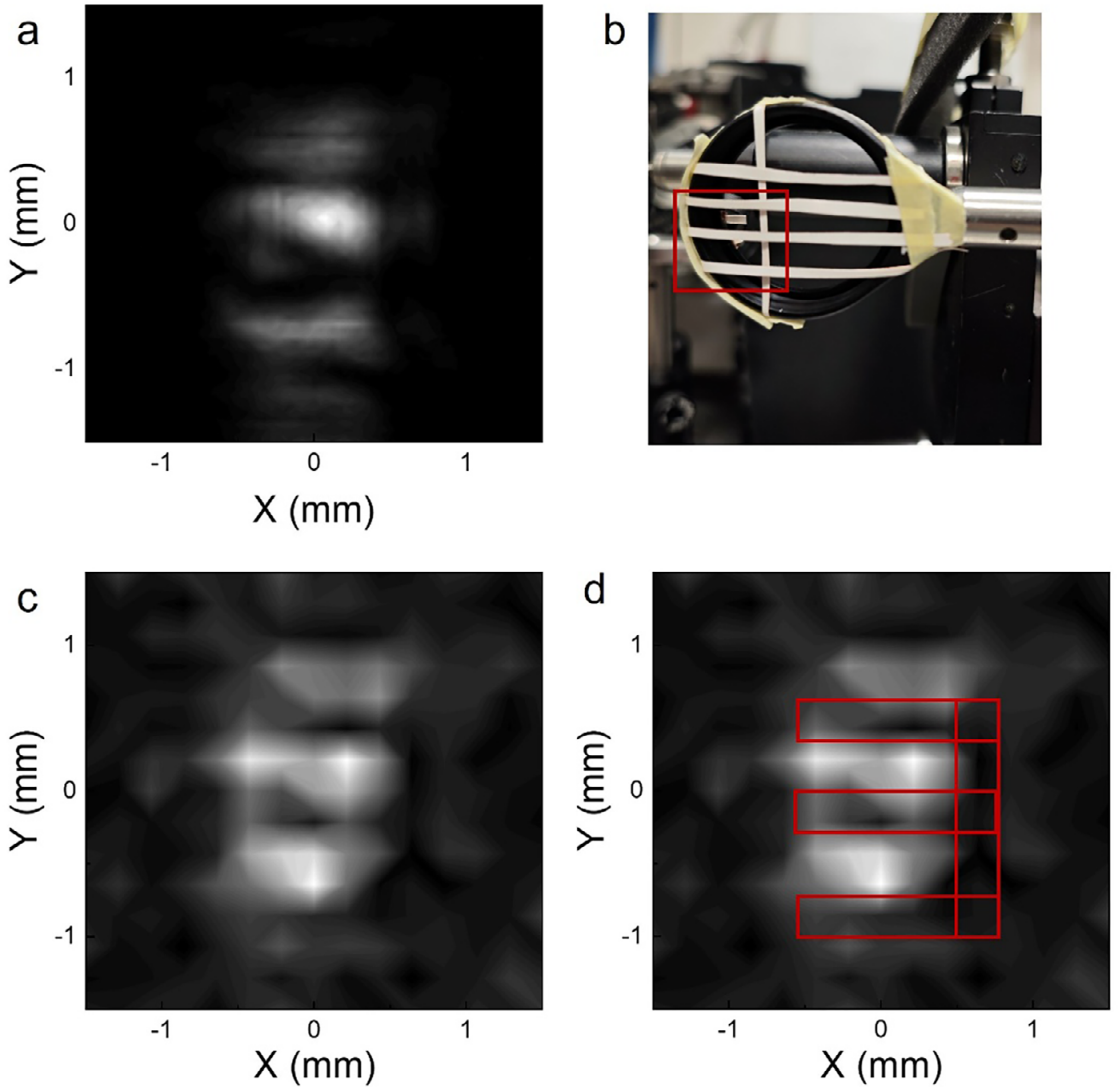
Image of an “E” shape object. (a) Imaging result of (b) with a single pixel raster scan. (b) “E” shape object made of copper. (c) Imaging result of (b) with the array. (d) Indication of the object in (c).

## 4 Conclusion

Building upon previous advances in graphene‐based THz detectors, we have successfully demonstrated the large‐area, scalable fabrication of THz cameras, with two distinct architectures: a linear‐array camera and a square array camera. By utilizing a QCL as a coherent THz illumination source, we systematically characterized the imaging performance of these detector arrays.

The implementation of robust signal processing techniques—including noise floor correction and dynamic signal normalization—allows to accurately reconstruct the spatial profile of the QCL beam and to demonstrate the efficacy of the system for imaging real‐world objects with high fidelity.

At the present stage of development, the demonstrated system is best suited for THz beam profiling, alignment, and calibration tasks, while its extension toward real‐time, high‐resolution focal‐plane imaging will require larger pixel counts, incoherent illumination, and fully parallel readout architectures.

Importantly, the fabrication methods and imaging protocols developed in this work are inherently compatible with extension to larger array formats, representing a concrete step toward ultra‐large‐area THz imaging systems. In this context, the term “scalable” refers to the underlying device architecture and fabrication strategy—namely, monolithic, lithographically defined arrays of individually addressable graphene pixels—rather than to the specific array size or imaging performance demonstrated at this stage. Achieving the transition toward fully parallel focal‐plane operation will require the integration of on‐chip biasing and readout circuitry, together with co‐design strategies that jointly optimize graphene‐based detector architectures and complementary integrated circuit technologies.

Furthermore, the described platform is intrinsically compatible with other emerging quantum materials, such as topological insulators, thereby extending its potential applicability to a broader class of optoelectronic systems. Continued progress in chemical vapor deposition (CVD) techniques to produce uniform, high‐quality graphene films will be critical for minimizing device‐to‐device variability and ensuring consistent large‐scale performance. Additionally, advancements in array architecture, fabrication precision, and system‐level control will be pivotal for transitioning graphene‐based THz imaging from laboratory demonstrations to practical, deployable technologies.

## 5 Materials and Methods

### 5.1 Fabrication

Monolayer graphene synthesized by chemical vapor deposition (CVD) was transferred onto SiO_2_/Si substrates using water‐assisted transfer [66]. The employed silicon substrates exhibited a resistivity >20 kΩ·cm. The transferred graphene/substrate stacks were left to dry overnight in a chemical hood. Subsequently, the samples were placed in a desiccator (1 h, pressure ~1 mBar) to further remove residual water and bubbles. Optical microscopy was employed to inspect the presence of visible blisters beneath the graphene layer. The dried devices were then annealed on a hot plate at 150°C for 1 h. After cooling, the samples were immersed in acetone, pre‐heated in a 50°C water bath, for 1 h to remove the PMMA support layer. The samples were subsequently transferred into isopropanol at room temperature and soaked for another hour.

Prior to device fabrication, the samples were annealed in vacuum at 250°C for 2 h. Graphene channels were patterned using electron‐beam lithography (EBL), followed by O_2_ plasma etching (O_2_ flow rate: 100 sccm, pressure: 0.06 mbar, RF power: 20 W) to remove the unwanted graphene regions. Source and drain electrodes were defined by EBL and fabricated via thermal evaporation of Cr/Au (5 nm/40 nm) followed by a lift‐off process. The dielectric layer was then defined by EBL, and 32 nm of HfO_2_ was deposited using atomic layer deposition (ALD). Bow‐tie antennas and extended electrodes for external circuitry were patterned by EBL and fabricated via thermal evaporation of Cr/Au (5 nm/100 nm), followed by lift‐off. Finally, external bonding pads were defined via laser direct writing, and Cr/Au (10 nm/200 nm) electrodes were deposited by thermal evaporation and lift‐off to complete the fabrication of the THz camera devices.

### 5.2 Readout Scheme

The fabricated multi‐pixel terahertz camera chip was mounted onto a custom‐designed multi‐channel printed circuit board (PCB). Electrical connections between the chip electrodes and the PCB were established via aluminum wire bonding. Multiplexed readout was implemented using a multi‐channel input, single‐channel output analog multiplexer (Texas Instruments, V599). As the multiplexer requires digital control signals to select input channels, an Arduino UNO R3 development board was employed to define the control logic through its digital output pins (S0, S1, S2, S3). The output signal from the multiplexer was first amplified by a low‐noise preamplifier, followed by lock‐in detection. In addition, since the V599 multiplexer can be enabled or disabled via an EN control pin, and the Arduino board provides multiple independent digital outputs, a single Arduino can, in principle, control two multiplexers simultaneously. This configuration enables the scalable readout of more than 16 pixels within the terahertz camera array.

## Conflicts of Interest

The authors declares no conflicts of interest.

## Supporting information




**Supporting File**: smtd70657‐sup‐0001‐SuppMat.docx.

## Data Availability

The data that support the findings of this study are available from the corresponding author upon reasonable request.
