# Supplementary material for: Scalable Room‐Temperature Terahertz Graphene Cameras
Source: Small Methods. 2026 Apr 27;10(21):e70657. doi: 10.1002/smtd.70657 (PMC13745982; doi:10.1002/smtd.70657)
Supplement: Supplementary file 1 [file SMTD-10-e70657-s001.docx]

**Supplementary Information**

**Scalable room-temperature terahertz graphene cameras**

Lili Shi^1^, Leonardo Viti^1^ and Miriam S. Vitiello^1^

*^1^NEST, CNR-Istituto Nanoscienze and Scuola Normale Superiore, Piazza San Silvestro 12, 56127 Pisa, Italy*

**1. Characterization of squared array camera**

Figure S1 displays the plot of the graphene channel resistance (*R*)as a function of the gate voltage (*V*_g_), measured on individual elements of the square-array photodetector, shown as a matrix reproducing the geometrical schematics of the pixel assembly in the tested cameras. For *V*_g_ varying from -2 V to 2 V, *R* varies between 20 kΩ and 48 kΩ, with the charge neutrality point (Dirac point) consistently located around *V*_g_ = -0.5 V, as illustrated in Figure S2a. Additionally, a negative correlation is observed between carrier mobility (*μ*) and residual carrier concentration (*n*_0_) (Fig. S2b).

**Figure S1. Summary of the channel resistance of square array camera. a** The resistance of the graphene channel versus gate voltage in the 5×5 squared array camera shown as a matrix reproducing the geometrical schematics of the pixel assembly in the tested cameras. Data from Dev3_3 and Dev3_5 are not shown due to lack of operation. **b** Pixel arrangement of square array camera.

**Figure S2. Electrical performance of squared array camera.** **a** Extrapolated charge neutrality point in the different pixels. **b** Extracted mobility versus residual carrier density in individual pixels.

Figure S3 displays the responsivity (*R*_ν_) of each pixel as a function of *V*_g_, shown as a matrix reproducing the geometrical schematics of the pixel assembly in the tested cameras, following the same rational of Fig. S1. The achieved maximum *R*_ν_ ranges between 25 and 36 V/W in most of the pixels (87%), revealing good uniformity across the array. Notably, all pixels maintain the highest responsivity around *V*_g_ = 0.5 V, which supports the use of a unified gate voltage for device operation.

**Figure S3. Summary of responsivity of the square array camera.** Plot of the measured responsivity as a function of the applied gate voltage in the 5×5 squared array camera shown as a matrix reproducing the geometrical schematics of the pixel assembly in the tested cameras. Data from Dev3_3 and Dev3_5 are not shown due to lack of operation.

Figure S4 illustrates the responsivity map of device 5_4, measured as a function of both left (V_GL_) and right (V_GR_) gate voltages. Six distinct regions emerge, corresponding to sign changes in the photoresponse. These regions are influenced by the non-monotonic variation of the Seebeck coefficient as a function of carrier density in the graphene channel. The polarity reversals occurring along the three dashed lines result in a six-fold pattern, which unequivocally identifies the photo-thermoelectric effect as the dominant detection mechanism in the p-n junction device.

**Figure S4. Thermoelectric response.** Color map of voltage responsivity (R_v_) as a function of V_GL_ and V_GR_. R_v_ undergoes polarity changes along the three dashed lines, corresponding to transitions between the different configurations of the *p–n* junction, attainable by polarizing the gates.

**2. Characterization of linear array camera**

Figure S5 displays the plot of *R* as a function of *V*_g_, measured on individual elements of the linear-array photodetector, shown as a matrix reproducing the geometrical schematics of the pixel assembly in the tested cameras. Different from the previous case, for *V*_g_ varying from -2 V to 1 V, *R* varies between 6 kΩ to 32 kΩ, exhibiting a relatively large fluctuation. The charge neutrality points (Dirac points) are located near –1.5 V and 1 V, as shown in Figure S6s, and display a much larger variability between individual pixel elements. Additionally, a negative correlation is observed between μ and *n*_0_ (Fig. S6b).

**Figure S5. Summary of the channel resistance of linear array camera.** **a** The resistance of the graphene channel versus gate voltage in the linear array camera shown as a matrix reproducing the geometrical schematics of the pixel assembly in the tested cameras. Data from the Dev10, Dev18, Dev19 and Dev20 are not shown due to lack of operation. **b** Pixel arrangement of linear array camera.

**Figure S6**. **Electrical performance of linear array camera. a** Extrapolated charge neutrality points in the different pixels. **b** extracted mobility versus residual carrier density in individual pixels.

Figure S7 displays *R*_v_ of each pixel as a function of *V*_g_, shown as a matrix reproducing the geometrical schematics of the pixel assembly in the tested cameras, following the same rational of Fig. S5. The achieved maximum *R*_v_ ranges between 6 and 10 V/W in most of the pixels (82%), revealing good uniformity across the array. Notably, all pixels maintain the highest responsivity around *V*_g_ = 1 V, providing a reliable basis for unified gate voltage control.

**Figure S7.** **Summary of responsivity of the linear array camera.** Plot of the measured responsivity as a function of the applied gate voltage in the linear array camera shown as a matrix reproducing the geometrical schematics of the pixel assembly in the tested cameras. Data from the Dev10, Dev18, Dev19 and Dev20 are not shown due to lack of operation.

**3. x-y scanning**

Figures S8 and S9 shows the imaging of the QCL beam obtained by scanning the square-array camera along the *x* and *y* directions, respectively, as schematically illustrated in Fig. 9c. When the camera is translated along the *x*-axis, the penultimate row of pixels is sequentially "activated" from left to right. In contrast, during *y*-direction scanning, the third column from the left is successively activated from top to bottom. These results demonstrate the camera’s ability to perform spatially resolved imaging through controlled lateral displacements in two orthogonal directions. The successful acquisition of images through both *x*- and *y*-directional scanning highlights the feasibility of two-dimensional raster scanning, offering a pathway toward comprehensive 2D image reconstruction over a defined area.

**Figure S8. *x*-scan of the QCL beam through the square array camera.** During the camera’s motion along the *x*-axis, pixels on the second-lowest row are sequentially illuminated from **a** to **d**.

**Figure S9. *y*-scan of the QCL beam through the square array camera.** During the camera’s motion along the *y-*axis, pixels on the third column are sequentially illuminated from **a** to **d**.

**4. Pixel Response Time**

To assess the response time of the SLG-based photodetectors, we conducted transient photoresponse measurements on representative single elements within the array. In this experiment, the output from a single pixel was directed to a fast transimpedance amplifier (TIA, DHPCA-100, Femto, Germany), and the TIA output was monitored using an oscilloscope (MSO64B, Tektronix). To ensure a sufficient signal-to-noise ratio (SNR) for the measured waveforms, we configured the TIA with the following settings: bandwidth of 3.5 MHz and a gain of 10^6^ V/A. The recorded oscilloscope trace is shown in figure S10, alongside the negative voltage pulse that drives the quantum cascade laser (QCL). By fitting the decay time (τ) of the oscilloscope trace with the exponential expression V_osc_ = c_1_ + V_0_ · exp(−(t − c_2_)/τ), where c_1_, c_2_, V_0_, and τ serve as fitting parameters. The fit converges for c_1_ = −29.0 ± 0.5 mV, c_2_ = 1.0 ± 0.001 μs, V_0_ = 234 ± 1 mV, and τ = 60 ± 1 ns. We note that the extracted time constant is robust against variations in the fitting window and initial parameter values, indicating that the observed decay reflects a genuine system response rather than random noise fluctuations. Furthermore, τ corresponds to a bandwidth of 2.6 MHz, very similar to the speed limit set by the TIA. This close correspondence demonstrates that the measured decay is dominated by the electronic bandwidth of the readout chain, rather than by the intrinsic response time of the graphene detector. Consequently, the retrieved response time is set-up limited.

**Figure S10. Response time analysis.** Time-resolved photodetection trace (black line), compared with the respective QCL voltage signal (azure line). The QCL pulse width is 1 μs. The onset of the photoresponse is affected by QCL power fluctuations, but the decay time (τ) can be fitted by an exponential function. We obtain τ = 60 ± 1 ns.

**5. Pixel Switching time**

A key feature of the current experimental setup is the straightforward pixel-by-pixel readout and multiplexing scheme, which is controlled by an Arduino board. The use of long integration times is not determined by the response time of individual pixel elements, but rather by the on-off switching of the multiplexer system, which induces photovoltage fluctuations during pixel transitions. In this context, we emphasize the significant potential for improvement. From a circuit perspective, the multi-pixel system could be co-integrated with a CMOS readout. This integration would allow each pixel at the analog front end to incorporate an instrumentation amplifier, utilizing dynamic offset cancellation techniques such as correlated double sampling (CDS) and chopper stabilization (CHS) [1]. Additionally, at the back end, oversampling and noise shaping through Σ-Δ modulation could be employed to shift quantization noise outside the band of interest [2], either in conjunction with or as an alternative to standard lock-in techniques. Importantly, this would allow for parallel pixel interrogation.

**6. Key Performance Indicators**

In this section, we provide a comparison between the key figures of merit of the GFET-based THz arrays and commercially available CMOS technology.

1. Responsivity and NEP: Our devices show responsivity on the order of tens to hundreds of V/W and NEP in the range of 10⁻^12^–10⁻¹⁰ W/√Hz, competitive with room-temperature CMOS THz cameras (NEP <10⁻⁹ W/√Hz).
2. Operating conditions: Graphene PTE detectors operate at room temperature without additional cooling, like CMOS devices, but their nanoscale active area allows for potential on-chip integration with planar antennas and multiplexed readout.
3. Integration and array capability: while CMOS technology benefits from mature large-pixel arrays and integrated readout electronics, graphene PTE detectors offer complementary advantages in ultrafast response and broadband detection, which could be exploited in future high-speed THz imaging arrays.

We believe this comparison clarifies the relative strengths of graphene PTE detectors and positions our work within the context of existing CMOS-based technologies.

| Technology (uncooled) | Frequency band (THz) | NEP (pW/Hz^1/2^) | Response time (ns) | Pixel pitch (um) | Cost |
| --- | --- | --- | --- | --- | --- |
| CMOS^[r1]^ | 0.1-4.3 THz^[r1, r2,r9]^ | 17-300^[r1,r3]^ | <1μs^[r4]^ (25 fps)^[r11]^ | 210^[r1]^ | >8000 $/mm^2 [r5]^ |
| Graphene PTE Detectors^[r6]^ | 0.1-4 THz Typical ^[r6]^ | 80-100^[r6,r7,r8,r10]^ | <100 (even <1 ns)^[r6]^ | 200[This work] | n.a. |

**Table I.** Comparison between CMOS based THz Camera and Graphene PTE based camera

**7. I-V characterization and photo-response**

Figure S11 shows the gate-voltage-dependent electrical transport and THz photo-response of the graphene PTE detector. The source–drain current *I*_sd_ exhibits a pronounced minimum near *V*_g_≈0 V, corresponding to the Dirac point of graphene, where the carrier density reaches its minimum. On either side of the Dirac point, the current increases with gate voltage, indicating a transition from p-type to n-type conduction and demonstrating effective electrostatic gating of the graphene channel.

The THz responsivity displays a strong dependence on the gate voltage, with a rapid increase occurring around *V*_g_≈−1 V, followed by a saturation at higher gate voltages. At the Dirac point, the sign reversal of the PTE response reflects the transition from hole-dominated to electron-dominated transport, accompanied by a corresponding change in the sign of the graphene Seebeck coefficient.

**Figure S11. Performance analysis.** Typical I-V curve and responsivity of the detector with *V*_g_ ranging from -2 to 2 V.

**8. Responsivity Normalization and Effective Area**

An important aspect in evaluating the optical performance of THz photodetectors is the normalization of the responsivity, particularly in the far-infrared regime where the detector dimensions are typically much smaller than both the radiation wavelength and the beam spot size. In such conditions, the estimation of the optical power coupled to the detector requires the definition of an effective area, A_eff_.

The coupled power can be expressed as P_a_= P_t_×(A_eff_/S_t_), where P_t_ is the total incident power and S_t_ is the beam spot area at the detector plane. Importantly, the extracted responsivity scales inversely with A_eff_; therefore, smaller effective areas lead to larger responsivity values, while larger effective areas provide more conservative estimates.

To assess the impact of the normalization choice, we consider three commonly used definitions of A_eff_:

**(i) Physical antenna area.**
The physical area of the bow-tie antenna is estimated by considering a circular area with diameter equal to the antenna length. For the antennas used in this work, this yields A_eff_≈1390 μm^2^. Normalization using this area provides the largest responsivity values and therefore represents the least conservative estimate.

**(ii) Effective area derived from antenna directivity.**
The antenna effective area can be expressed as A_eff_=D_0_λ^2^/(4π), where D_0_ is the antenna directivity in the direction of illumination. For bow-tie antennas of similar geometry and under identical experimental conditions, previously reported measurements yield D_0_≈3.75, resulting in A_eff_≈3300 μm^2^.

**(iii) Diffraction-limited area.**
The diffraction-limited area S_λ_=λ^2^/4 represents the minimum physically achievable spot size for free-space radiation at numerical aperture NA ≈ 1. At 2.9 THz, this yields S_λ_≈2800 μm^2^.

Notably, the diffraction-limited area is comparable to the directivity-based effective area (difference <15%), while remaining significantly larger than the physical antenna area. As a result, normalization using S_λ_ provides a conservative and physically well-defined estimate of responsivity that avoids overestimation and does not require direct measurement of antenna radiation patterns or complex electromagnetic simulations, which are often sensitive to bonding pads, wiring, and substrate effects.

For these reasons, and in line with established practice in THz detector characterization, we adopt the diffraction-limited area as the reference normalization throughout this work.

**9. Comparison with other terahertz cameras**

Similar to section 6, we also list the comparison between other techniques and our work as shown in Table II:

1. Responsivity and NEP: Our devices show responsivity on the order of tens to hundreds of V/W and NEP in the range of 10⁻^12^–10⁻¹⁰ W/√Hz, competitive with other techniques (NEP <10⁻⁹ W/√Hz).
2. Responsivity: Although graphene PTE camera shows lower responsivity, but it can work under higher THz frequency and operate without bias.
3. Integration and array capability: Compared to other techniques, the graphene PTE camera just require planar lithography which is easier than other techniques.

| **Performance** | **HEMT[r11]** | **Schottky diode [r12]** | **MEMS/NEMS[r13]** | **Graphene PTE [This work]** |
| --- | --- | --- | --- | --- |
| NEP (nW/Hz^1/2)^ | <0.1 | <0.1 | 0.01-100 | <1 |
| Responsivity | 20 mA/W @1 THz | 1100V/W @ 1THz | ~5×10^3^ V/W@2.5 THz | 35 V/W @ 2.86 THz |
| Operation frequency (THz) | 0.1-1.5 | 0.01-1.5 | 0.01-100 | 0.1-IR |
| Integration | Requires III–V materials; heterogeneous or backend integration needed | Mature technology; integration complexity increases for dense arrays | High sensitivity but complex fabrication and packaging | Monolithic integration feasible; performance limited by CVD graphene uniformity |
| Scalability | Moderate | Moderate | Limited | High (planar, lithography-defined) |
| Bias | Yes | Yes | Yes | No |

**Table II.** Comparison between HEMT, Schottky diode, MEMS/NMES and Graphene PTE based camera

**References**

[1]. J. Kang, W. Han, S. Kim and J. Jun, "Comprehensive Analysis of Design and Performance Evaluation Methodologies for a High-Resolution Read-Out IC With Ultralow 1/ f Corner for DC Measurement Applications," in *IEEE Transactions on Instrumentation and Measurement*, vol. 74, pp. 1-14, 2025, Art no. 2003914, doi: 10.1109/TIM.2025.3548195.

[2] J. -S. Huang, S. -C. Kuo and C. -H. Chen, "A Multistep Multistage Fifth-Order Incremental Delta Sigma Analog-to-Digital Converter for Sensor Interfaces," in *IEEE Journal of Solid-State Circuits*, vol. 58, no. 10, pp. 2733-2744, Oct. 2023, doi: 10.1109/JSSC.2023.3288198.

List of references for Table I:

[r1] Schuster, Franz, et al. "Broadband terahertz imaging with highly sensitive silicon CMOS detectors." *Optics express* 19.8 (2011): 7827-7832.;

[r2]Lin, Yue, et al. "Focal plane imaging system based on CMOS terahertz detector." *Journal of Physics: Conference Series*. Vol. 2806. No. 1. IOP Publishing, 2024.

[r3]Hasan, Md Soyaeb, et al. "Terahertz radiation detectors using CMOS compatible SOI substrates." *Advanced Functional Materials* 34.41 (2024): 2400313.

[r4]Zagrajek, Przemyslaw, et al. "Time resolution and dynamic range of field-effect transistor–based terahertz detectors." *Journal of Infrared, Millimeter, and Terahertz Waves* 40.7 (2019): 703-719.

[r5]https://www.cmc.ca/tsmc-65-nm-gp-cmos/

[r6]Cai, Xinghan, et al. "Sensitive room-temperature terahertz detection via the photothermoelectric effect in graphene." Nature nanotechnology 9.10 (2014): 814-819.

[r7]Liu, Jianlong, et al. "Recent progress in the development of graphene detector for terahertz detection." *Sensors* 21.15 (2021): 4987.

[r8]Yao, Yifan, et al. "Configurable microcavity-enhanced graphene photothermoelectric terahertz detectors." *Photonics Research* 12.10 (2024): 2300-2310.

[r9] A. Lisauskas et al., "Terahertz detection and coherent imaging from 0.2 to 4.3 THz with silicon CMOS field-effect transistors," 2012 IEEE/MTT-S International Microwave Symposium Digest, Montreal, QC, Canada, 2012, pp. 1-3, doi: 10.1109/MWSYM.2012.6259599

[r10] Liu, J.; Li, X.; Jiang, R.; Yang, K.; Zhao, J.; Khan, S.A.; He, J.; Liu, P.; Zhu, J.; Zeng, B. Recent Progress in the Development of Graphene Detector for Terahertz Detection. Sensors 2021, 21, 4987. https://doi.org/10.3390/s21154987

List of references for Table II:

[r11]A. Rämer et al. "Monolithically Integrated THz Detectors Based on High-Electron-Mobility Transistors." Sensors 25.11 (2025): 3539.

[r12]<https://www.toptica.com/products/terahertz-systems/accessories/schottky-receivers/>

[r13]L.Chao, Y. Zhang, and K. Hirakawa. "Terahertz detectors using microelectromechanical system resonators." Sensors 23.13 (2023): 5938.
